# Systematic search for enhancer elements and somatic allelic imbalance at seven low-penetrance colorectal cancer predisposition loci

**DOI:** 10.1186/1471-2350-12-23

**Published:** 2011-02-14

**Authors:** Iina Niittymäki, Sari Tuupanen, Yilong Li, Heikki Järvinen, Jukka-Pekka Mecklin, Ian PM Tomlinson, Richard S Houlston, Auli Karhu, Lauri A Aaltonen

**Affiliations:** 1 Department of Medical Genetics, Genome-Scale Biology Research Program, Biomedicum Helsinki, University of Helsinki, Helsinki, Finland; 2 Department of Surgery, Helsinki University Hospital, Helsinki, Finland; 3 Department of Surgery, Jyväskylä Central Hospital, Jyväskylä, Finland; 4 Molecular and Population Genetics, Wellcome Trust Centre for Human Genetics, and Comprehensive Biomedical Research Centre, Oxford, UK; 5 Section of Cancer Genetics, Institute of Cancer Research, Sutton, UK

## Abstract

**Background:**

Common single-nucleotide polymorphisms (SNPs) in ten chromosomal loci have been shown to predispose to colorectal cancer (CRC) in genome-wide association studies. A plausible biological mechanism of CRC susceptibility associated with genetic variation has so far only been proposed for three loci, each pointing to variants that affect gene expression through distant regulatory elements. In this study, we aimed to gain insight into the molecular basis of seven low-penetrance CRC loci tagged by rs4779584 at 15q13, rs10795668 at 10p14, rs3802842 at 11q23, rs4444235 at 14q22, rs9929218 at 16q22, rs10411210 at 19q13, and rs961253 at 20p12.

**Methods:**

Possible somatic gain of the risk allele or loss of the protective allele was studied by analyzing allelic imbalance in tumour and corresponding normal tissue samples of heterozygous patients. Functional variants were searched from *in silico *predicted enhancer elements locating inside the CRC-associating linkage-disequilibrium regions.

**Results:**

No allelic imbalance targeting the SNPs was observed at any of the seven loci. Altogether, 12 SNPs that were predicted to disrupt potential transcription factor binding sequences were genotyped in the same population-based case-control series as the seven tagging SNPs originally. None showed association with CRC.

**Conclusions:**

The results of the allelic imbalance analysis suggest that the seven CRC risk variants are not somatically selected for in the neoplastic progression. The bioinformatic approach was unable to pinpoint cancer-causing variants at any of the seven loci. While it is possible that many of the predisposition loci for CRC are involved in control of gene expression by targeting transcription factor binding sites, also other possibilities, such as regulatory RNAs, should be considered.

## Background

Ten chromosomal loci have thus far been shown to modestly increase colorectal cancer (CRC) risk, based on genome-wide association studies (GWASs) [1-9]. The tagging single-nucleotide polymorphisms (SNPs) with the strongest association signal in each locus were rs6983267 at 8q24 [1-3], rs4939827 at 18q21 [4], rs4779584 at 15q13 [5], rs16892766 at 8q23 [6], rs10795668 at 10p14 [6], rs3802842 at 11q23 [7,8], rs4444235 at 14q22 [9], rs9929218 at 16q22 [9], rs10411210 at 19q13 [9], and rs961253 at 20p12 [9]. Each of the ten loci independently predispose to CRC with allelic odds ratios (ORs) of <1.3 and risk allele frequencies range between 7-90% in the general population [10].

GWASs are based on genotyping SNPs which tag linkage disequilibrium (LD) blocks in the genome, thus capturing a high proportion of common genetic variation. Hence, usually the associating tag SNPs are not themselves causal but rather are in LD with disease-causing variants. We have previously demonstrated that the tag SNP rs6983267 at 8q24 directly disrupts a TCF-4 transcription factor binding site and enhances Wnt signalling in the colon [11]. This was supported by a simultaneous study showing a physical interaction between rs6983267 region and *MYC *proto-oncogene [12]. Furthermore, allelic imbalance (AI) at 8q24 seen in colorectal tumours favors the risk allele G of rs6983267, suggesting that the locus is somatically selected for in tumourigenesis [13]. At 18q21, a novel variant that correlates with rs4939827 reduces *SMAD7 *expression, leading to aberrant TGFβ (transforming growth factor beta) signalling [14]. Finally, rs16888589 at the 8q23 CRC locus was shown to influence *EIF3 H *expression by physically interacting with the promoter [15]. Unlike at 8q24, no significant difference in the alleles targeted by imbalance was detected in rs4939827 at 18q21 [4,14] nor in rs16892766 at 8q23 [15]. It is therefore likely that many low-penetrance cancer susceptibility loci may be explained by subtle changes in distant regulatory elements, and these changes can also play a role in the somatic tumour development. Based on the genes that locate inside or near the CRC-associating regions, including *GREM1 *at 15q13 and *BMP4 *at 14q22, alterations in TGFβ-superfamily signalling appear to be at the basis of several loci [10].

Possible biological mechanisms underlying CRC predisposition are yet to be discovered in the seven low-penetrance loci at 15q13, 10p14, 11q23, 14q22, 16q22, 19q13, and 20p12, which is the focus of this study. First, possible somatic selection of the risk alleles was evaluated in heterozygous individuals by examining the tagging SNPs in tumour and corresponding normal tissues. The location of the SNPs at predicted enhancer elements was then investigated with an *in silico *tool. As none of the tagging SNPs were predicted to locate at transcription factor binding sites, the analysis was extended to the given LD regions. Putative functional variants were searched by genotyping all the known SNPs inside the associating LD regions that were predicted to disrupt transcription factor binding sites.

## Methods

### Study population

A population-based series of 1 042 CRC samples collected since 1994 from nine Finnish central hospitals was used in this study [16,17]. Both germline DNA extracted from blood or normal colonic tissue and corresponding fresh-frozen tumour DNA were available. Information on histological tumour grade and Duke's stage was obtained from pathology reports. The 837 control DNA samples used in this study were anonymous healthy blood donors from the Finnish Red Cross Blood Transfusion Service. Samples and clinicopathologic data were obtained with informed consent and ethical review board approval in accordance with the declaration of Helsinki.

### Allelic imbalance (AI) analysis

Allelic ratios were compared by sequencing tumour and respective normal tissue DNA in heterozygous patients, as described previously [13,18,19]. In brief, allele peak height ratios of <0.6 and >1.67 between normal and tumour samples were considered imbalance (Tumour^(Allele1/Allele2)^/Normal^(Allele1/Allele2)^). Tumour and normal samples were sequenced using Applied Biosystems BigDye v3.1 sequencing chemistry and ABI3730 Automatic DNA sequencer (Applied Biosystems, Foster City, CA, USA). Peak heights were manually measured from sequencing chromatograms using Chromas http://www.technelysium.com.au and Sequence Scanner (Applied Biosystems) softwares, based on which the allelic ratio was calculated. First, 90 tumour-normal pairs were analyzed. If any trend towards imbalance was observed, all the available heterozygote samples were analyzed. All the tumours were microscopically evaluated by a pathologist and at least 64% of the analyzed tumours contained ≥70% of carcinoma tissue.

### Identification of SNPs at transcription factor binding sites

A computational tool called Enhancer Element Locator (EEL) [20,21], that aligns genomic sequence from two species and predicts the location of putative transcription factor binding sequences and enhancer elements, was used. In the output, a score is given to each element based on conservation, clustering, and predicted affinity of the binding sites. One Mb of human and corresponding mouse sequence surrounding each SNP (500 kb of flanking sequence up- and downstream) was exported from the Ensembl database vs 54 http://www.ensembl.org/index.html. Transcription factor binding-affinity matrices from the publicly available Jaspar database http://jaspar.genereg.net/ and those published elsewhere were used in the alignment [22-24], that was done with default parameters. All the known SNPs that were predicted to locate directly at transcription factor binding sites were selected from enhancers that were inside the CRC-associating LD regions. We defined LD blocks using HapMap data http://hapmap.ncbi.nlm.nih.gov/: chr15: 30 782 050 - 30 841 010 bps (59 kb; human genome build 36) in rs4779584 [5], chr10: 8 730 000 - 8 810 000 bps (80 kb) in rs10795668 [6], chr11: 110 640 000 - 110 690 000 bps (50 kb) in rs3802842 [8], chr14: 53 477 192 - 53 494 200 bps (17 kb) in rs4444235 [9], chr16: 67 286 613 - 67 396 803 bps (110 kb) in rs9929218 [9], chr19: 38 203 614 - 38 300 573 bps (97 kb) in rs10411210 [9], and chr20: 6 316 089 - 6 354 440 bps (38 kb) in rs961253 [9]. The analysis was restricted to such SNPs where the EEL score for the given element was ≥ 300.

### Genotyping of SNPs in cases and controls

Genotyping was carried out using Sequenom MassArray iPlex Gold (Sequenom, San Diego, CA, USA) performed by the Institute for Molecular Medicine Finland FIMM Technology Centre, University of Helsinki. Each 96-well sample plate contained two negative water controls and two positive CEPH controls. The concordance between duplicate controls was 99,79% (479/480 genotypes). Twelve SNPs (rs11631292, rs62002613, rs17485426, ENSSNP10169878, rs1999638, rs12273224, rs45615536, rs10505287, rs57897735 rs10505283, rs2761880, and rs12893484) were successfully genotyped with MassArray. Three remaining SNPs (rs28768389, rs12899808, and rs34812868) were genotyped by direct genomic sequencing using Applied Biosystems BigDye v3.1 sequencing chemistry and ABI3730 Automatic DNA sequencer (Applied Biosystems).

### Statistical analysis

All the analyses were performed with R software. Exact binomial test was used in allelic imbalance analysis. Allelic odds ratios, 95% confidence intervals, and *P*-values were calculated with Pearson's Chi-squared test. To adjust for multiple testing we applied a Bonferroni correction (not shown in Table 1). Fisher's exact test was used in the analysis of clinicopathological characteristics.

**Table 1:** Genotyping of 12 SNPS that locate in transcription factor binding sites in low-penetrance CRC loci

*Locus*	SNP bp*	*SNP ID*	*MAF controls*	*MAF cases*	*OR*	*95% CI*	*P*	*Cases (n)*	*Controls (n)*	Binding sequence***	*TF*
*15q13*	*30 782 048*	rs4779584**	*0.295*	*0.323*	*1.14*	*1.00 - 1.31*	*0.05*	*981*	*1 024*		

	30 806 855	rs28768389	0.088	0.073	1.24	0.97-1.57	0.08	976	837	AAYCAATCACATA	GATA2-FOXL1
	30 824 927	rs11631292	0.123	0.108	1.16	0.95-1.42	0.15	1 009	823	YATCC	GATA2
	30 825 051	rs62002613	*monomorphic*					1 011	824	KATCT	GATA2
	30 806 691	rs12899808	*monomorphic*					976	837	MCAACAT	BRCA1
	30 804 840	rs34812868	0.431	0.402	1.13	0.99-1.29	0.08	913	821	GTRTCT	GATA1
											
*10p14*	*8 741 225*	rs10795668**	*0.307*	*0.271*	*1.19*	*1.04-1.37*	*0.01*	*951*	*983*		

	8 743 013	rs17485426	0.129	0.137	1.07	0.88-1.29	0.51	1 010	824	TATMTAAA	FOXL1
	8 742 714	ENSSNP10169878	0.129	0.137	1.07	0.88-1.29	0.51	1 010	824	CAATCTTAWTTTTT	GATA3-FOXC1
	8 783 743	rs1999638	0.123	0.136	1.12	0.92-1.37	0.24	1 010	824	RAATCT	GATA3
											
*11q23*	*110 676 919*	rs3802842**	*0.239*	*0.276*	*1.22*	*1.06-1.40*	*0.01*	*970*	*994*		

	110 645 918	rs12273224	*monomorphic*					997	823	TAAATRTA	FOXL1
	110 669 934	rs45615536	0.01	0.006	1.63	0.77-3.57	0.19	1 009	824	TKGGGG	MZF1
											
*14q22*	*53 480 669*	rs4444235**	*0.426*	*0.462*	*1.16*	*1.02-1.33*	*0.03*	*933*	*828*		

	53 488 736	rs2761880	0.009	0.008	1.08	0.51-2.23	0.85	1 011	824	GTGTTTYTTTATTCTT	FOXJ3
	53 484 488	rs12893484	0.384	0.414	1.13	0.99-1.29	0.07	1 010	824	GCCRCAGGC	TFAP2A

* Human genome build 36; **Tag SNPs genotyped in previous studies [6,8,9]; *** SNPs indicated as Y = C/T, K = G/T, M = A/C, R = A/G, W = A/T; MAF = minor allele frequency, TF = transcription factor; rs11631292, rs62002613, rs17485426, ENSSNP10169878, rs1999638, rs12273224, rs45615536, rs10505287, rs57897735 rs10505283, rs2761880, and rs12893484 were genotyped using Sequenom MassArray iPlex Gold; rs28768389, rs12899808, and rs34812868 were genotyped using genomic sequencing

## Results

The tag SNPs of seven low-penetrance loci were sequenced in heterozygous tumour-normal pairs, in order to detect possible AI occurring in the neoplastic progression. The risk alleles were not significantly targeted by AI in any of the seven SNPs (Table 2). The frequency of overall imbalance (loss of either the risk or the neutral allele) ranged between 9 and 31% at the seven loci (Table 2). In rs10411210, ten tumours showed loss of the neutral allele and five tumours loss of the risk allele, however AI occurred altogether in only 9% of the tumours (Table 2). No significant differences were observed between the two AI groups in terms of Duke's stage (*P *= 0.3) or histological grade (*P *= 1.0).

**Table 2:** Results of allelic imbalance analysis in low-penetrance CRC loci

*Locus*	*SNP*	*Risk allele*	*Neutral allele*	*Imbalance^a^*	*Loss neutral*	*Loss risk*	*P^b^*
15q13	rs4779584	T	C	17/87 (20%)	8	9	1
10p14	rs10795668	G	A	12/90 (13%)	6	6	1
11q23	rs3802842	C	A	9/89 (10%)	4	5	1
14q22	rs4444235	C	T	17/90 (19%)	7	10	0.63
16q22	rs9929218	G	A	10/90 (11%)	6	4	0.75
19q13	rs10411210	C	T	15/174 (9%)	10^c^	5^c^	0.30
20p12	rs961253	A	C	27/88 (31%)	11	16	0.44

*^a ^*Overall allelic imbalance per heterozygote tumour-normal pairs analyzed

*^b ^*Random targeting of alleles (exact binomial test)

^c ^Dukes A-B/C-D: Loss neutral 7/3, loss risk 2/3

The LD regions containing the seven tag SNPs were further analyzed with EEL. None of the seven tag SNPs located in predicted transcription factor binding sites. Thirteen other SNPs in the LD regions located at predicted binding sites in elements with a score ≥ 300. Three out of seven loci (16q22, 19q13, and 20p12) contained no SNPs at transcription factor binding sites in elements with a score ≥ 300. One of the SNPs, rs11853552 at 15q13, was already previously genotyped by Jaeger et al. (2008) [5], and was therefore excluded from the analysis. The remaining 12 SNPs were genotyped in the same Finnish case-control series as the tag SNPs in previous studies (Table 1) [6,8,9]. None of the SNPs showed association with CRC (Table 1).

Three of the 12 SNPs (rs28768389, rs12899808, and rs34812868 in 15q13) were genotyped using sequencing and four additional polymorphisms that did not locate in any predicted binding sites were observed in the sequencing fragments. One of these SNPs, rs35614970 (A6/A3), showed significant association with CRC (OR 1.2, 95% CI 1.03-1.38, *P *= 0.02). The frequency of A6 was 0.705 in controls and 0.741 in cases. This association did not remain significant after correction for multiple testing (*P *= 0.21, Bonferroni correction for 13 SNPs). None of the other additional SNPs (rs11071928, rs34944927, and a novel C to T change at chr15: 30 806 922) showed association with CRC.

## Discussion

In this study, we exploited the same approach as for 8q24 to systematically analyze molecular basis of seven low-penetrance CRC loci where the cancer-causing variants have not yet been identified. This is the first time, to our knowledge, that rs4779584, rs10795668, rs4444235, rs9929218, rs10411210, and rs961253 have been analyzed for possible AI in colorectal tumours. Sequencing of fresh-frozen tumour material provides accurate data on possible loss of the neutral allele or gain of the risk allele. In case of selective imbalance, subsequent copy number analyses can reveal whether the role of a variant resembles that of a tumour suppressor or an oncogene, and guide further functional efforts. The 8q24 locus, where gain of the risk allele was observed in rs6983267 [11,13], currently seems to be the only low-penetrance risk locus for CRC that is somatically enriched during tumourigenesis. Lack of imbalance favouring risk allele does not, however, preclude involvement in germline predisposition. It is therefore possible that some of the seven susceptibility variants analyzed in this study play a role in the early stages of neoplastic development, without providing further selective advantage in the somatic cancer progression.

Interestingly, 19q13 gain is common in both primary and metastatic CRC [25]. We did not, however, observe any significant difference in the alleles targeted by AI in rs10411210, nor association of neutral allele loss with more advanced disease stage. Loss of heterozygosity involving 10p14 has also been reported to occur in CRC [26] but we found no evidence of risk allele selection in rs10795668. Furthermore, deletion of 11q23-q24 is a frequent event in CRC, among other tumour types [27]. However, Tenesa et al. (2008) found no AI in favour of the risk allele in rs3802842 based on up to 43 CRCs [7], which is now confirmed by our analysis of 89 CRCs. Finally, although 18q loss is common in CRC [28], Broderick et al. (2007) observed no selection of the risk allele at rs4939827 in 248 CRC cases [4].

The location of rs6983267 at TCF-4 binding sequence was recently discovered using EEL [11], which prompted us to utilize this powerful tool also for the seven susceptibility loci. Transcription factor tissue specificities are incompletely understood, supporting the rationale of our unbiased approach of not restricting to colon-specific factors. Given that the tagging SNPs at the seven loci lie in noncoding regions, the most likely underlying mechanism is differential gene expression through enhancers or repressors. Although regulatory SNPs have been identified in the loci successfully fine-mapped so far, our study underscores the importance of considering also other mechanisms. For instance, sequence variation affecting noncoding regulatory RNAs, many of which have been linked to cancer-associated pathways, could explain some of the predisposition loci devoid of coding genes.

## Conclusion

While successful in the 8q24 locus, the approach used in this study was unable to pinpoint causal variants in the seven low-penetrance CRC loci analyzed. Finding the underlying functional changes in the GWAS loci is a challenging, yet important, task in order to fully understand the biology behind common CRC susceptibility.

## Competing interests

The authors declare that they have no competing interests.

## Authors' contributions

IN drafted the manuscript, and together with ST participated in the design, carried out the experiments and analyzed the data, YL carried out the EEL analysis, IPMT acquired data, RSH acquired data and helped in writing the manuscript, HJ and JPM acquired patient samples and data, AK and LA were involved in the design and coordination and helped in writing the manuscript. All authors read and approved the manuscript.

## Pre-publication history

The pre-publication history for this paper can be accessed here:

http://www.biomedcentral.com/1471-2350/12/23/prepub
